# Supplementary material for: A Prediction Model for Metachronous Peritoneal Carcinomatosis in Patients with Stage T4 Colon Cancer after Curative Resection
Source: Cancers (Basel). 2021 Jun 4;13(11):2808. doi: 10.3390/cancers13112808 (PMC8200190; doi:10.3390/cancers13112808)
Supplement: Supplementary file 1 [file cancers-13-02808-s001.zip › cancers-1164694-supplementary.pdf]

cancers

MDPI

Table S1. Assessment of proportional hazard assumption.

	Chi-squared Statistics	Degrees of Freedom	*p*-value
Tumor location	0.007	1	0.93
Preoperative CEA value	0.522	1	0.47
Histologic grade	0.873	1	0.35
Tumor stage	0.764	1	0.38
N1 stage	0.247	1	0.62
N2 stage	0.096	1	0.76
Global	2.261	6	0.89
